# Disease Clearance Reduces the Risk of Adverse Outcomes and Provides Additional Benefit Beyond Individual Clinical, Endoscopic, or Histological Remission in Ulcerative Colitis

**DOI:** 10.3390/jcm15020433

**Published:** 2026-01-06

**Authors:** Laura Ramos, Raquel de la Barreda, David Nicolás-Pérez, Manuel Hernández-Guerra, Enrique Quintero

**Affiliations:** 1 IBD Unit, Department of Gastroenterology, Hospital Universitario de Canarias. C/Ofra n/n, 38320 La Laguna, Spain; 2 Gastroenterology Department, Hospital Universitario de Canarias, 38320 La Laguna, Spain; rakelbh89@gmail.com (R.d.l.B.); dnicolasp@telefonica.net (D.N.-P.); mhernand@ull.edu.es (M.H.-G.); equinter@gmail.com (E.Q.); 3 Department of Internal Medicine, University of La Laguna, 38320 La Laguna, Spain

**Keywords:** ulcerative colitis, disease clearance, histological remission, endoscopic remission, clinical remission, outcome

## Abstract

**Background and Aims:** Recent advances in Ulcerative Colitis (UC) treatment have the potential to improve clinical outcomes if inflammation is controlled. The emerging concept of “disease clearance” (DC) aims to achieve combined clinical, endoscopic, and histological remission. However, whether DC is associated with better outcomes remains unclear. This study aimed to evaluate the impact of DC on UC patients. **Methods:** Consecutive UC patients who underwent colonoscopy and had clinical follow-up of at least 18 months were included between September 2012 and December 2017. Clinical condition (Patient-Reported Outcome, PRO), Mayo endoscopic score (MES), and histopathology (Geboes score, GS) were assessed at baseline and every 6 months. Disease clearance (DC) was defined as simultaneous clinical remission (PRO2 = 0), endoscopic remission (MES = 0), and histological remission (GS ≤ 2). **Results:** A total of 101 UC patients with a median time since diagnosis of 103 ± 102 months were included. At baseline colonoscopy, 52 patients (51.5%) achieved DC. These subjects had a lower rate of negative outcomes than those without DC (21.2% vs. 59.2%, *p* < 0.001). Kaplan–Meier curves confirmed a lower risk of clinical relapse in DC patients (log-rank, *p* = 0.001). Among patients with endoscopic remission, only those with DC had a lower risk of negative outcomes (21.2% vs. 75%, *p* < 0.01). Achieving DC reduced the risk of flare-ups even in patients with clinical remission or histological remission. **Conclusions:** Achieving DC in UC patients reduces the risk of negative outcomes during follow-up, supporting its inclusion as a key composite treatment goal in UC management.

## 1. Introduction

Ulcerative colitis (UC) is a chronic and progressive inflammatory bowel disease (IBD) that, if inadequately controlled, may progress to structural bowel damage, anorectal dysfunction, and an increased risk of colectomy and colorectal cancer [1,2]. In recent years, therapeutic strategies in UC have shifted from symptom management toward objective, inflammation-driven targets. The STRIDE-II initiative has defined clinical and endoscopic remission as primary goals of therapy, while histological remission is recognized as a desirable long-term objective [3].

Clinical remission, marked by normalized stool frequency and absence of rectal bleeding, and endoscopic remission, defined as a Mayo Endoscopic Subscore (MES) of 0, are both associated with improved outcomes, including reduced relapse, hospitalization, and surgical risk [4,5,6]. Nevertheless, histological activity may persist despite endoscopic healing and has been independently associated with adverse outcomes, highlighting the limitations of evaluating only superficial or symptomatic remission [7,8,9].

To address these challenges, the concept of disease clearance (DC) has emerged. DC is defined as the simultaneous achievement of clinical, endoscopic, and histological remission, reflecting a comprehensive approach that aims to achieve true control of intestinal inflammation [10,11,12]. This integrative strategy assumes that eliminating inflammation across all disease domains is essential for optimal disease management—including future molecular targets—and may modify the natural history of UC, improving long-term outcomes [11,13].

Inspired by therapeutic models in other immune-mediated diseases such as psoriasis, DC aspires to become the ultimate goal in UC management [14]. A recent international consensus has proposed a standardized definition of DC, and recent studies have shown that, although stringent, this target is achievable in a substantial proportion of patients and correlates with favorable clinical outcomes [11,15].

Despite growing support for DC as a treatment target, data on the feasibility of achieving DC in real-world practice and its impact on clinical outcomes in UC patients remain limited. Therefore, the objective of our study was to evaluate the impact of DC in a cohort of UC patients in a tertiary university hospital.

## 2. Material and Methods

### 2.1. Study Population

This was a prospective, single-center cohort study including patients with an established diagnosis of UC. Eligible patients were adults (≥18 years) with a confirmed diagnosis of UC for at least six months and under regular follow-up at an IBD referral unit, allowing for a minimum monitoring period of 18 months. Colonoscopy was indicated by the treating physician as part of routine clinical management, including evaluation of treatment response, assessment of disease activity for therapeutic decision-making, or colorectal cancer (CRC) screening. Exclusion criteria were inability to provide informed consent or refusal/inability to undergo colonoscopy.

### 2.2. Study Procedures and Data Collection

Patients were recruited through the Endoscopy Unit at the Hospital Universitario de Canarias. Informed consent was obtained before colonoscopy following a standardized explanation of the study objectives. At the time of colonoscopy, clinical activity was assessed using the Total Mayo Score (MayoT), which includes PRO2 parameters (stool frequency and rectal bleeding), the physician’s global assessment, and the Mayo Endoscopic Subscore (MES).

Endoscopic activity was documented using the MES. Biopsies were obtained from the most inflamed macroscopic area and histologically evaluated using the Geboes Score (GS), as per the institutional protocol. Medical records were reviewed to collect demographic and disease-related variables, including age, sex, UC extent (Montreal classification), and disease duration. Current treatment at the time of colonoscopy and clinical and treatment stability over the previous three months were also recorded.

### 2.3. Definition of Variables

Disease clearance (DC) was defined as the simultaneous achievement of:

- Clinical remission (CR): normalized stool frequency with absence of rectal bleeding (PRO2 = 0);
- Endoscopic remission (ER): MES = 0 (normal mucosal appearance);
- Histological remission (HR): GS ≤ 2 (absence of neutrophils in the mucosa).

Negative outcomes (flares or complications) were defined as clinical relapse requiring systemic or topical corticosteroids, treatment optimization or escalation, switching to a different therapeutic agent, hospitalization due to UC, or colectomy.

Based on the collected data, patients were classified into two groups: those achieving DC (meeting all three remission criteria) (DC group) and those not achieving DC (non-DC group). The non-DC group was further stratified according to endoscopic activity: endoscopic remission (MES = 0) vs. active disease (MES ≥ 1).

### 2.4. Follow-Up and Outcome Assessment

All patients were followed up for 18 months. Clinical evaluations were performed every six months to monitor flares or complications. The occurrence, timing, and management of these outcomes were systematically recorded throughout the follow-up period.

### 2.5. Ethical Considerations

The study was approved by the Ethics Committee of the Complejo Hospitalario Universitario de Canarias (registration number 285/2010). Written informed consent was obtained from all participants before colonoscopy.

### 2.6. Statistical Analysis

Continuous variables were presented as medians and interquartile ranges (IQRs), while categorical variables were expressed as frequencies and percentages. Comparisons between subgroups were made using the Chi-square or Fisher’s exact test for categorical variables, and Student’s *t*-test or Mann–Whitney U test for continuous variables, as appropriate. Logistic regression analysis was performed to identify predictors of adverse outcomes. Kaplan–Meier curves were constructed to assess flare-free or event-free survival between DC and non-DC groups. Statistical analyses were conducted using SPSS version 20 (IBM Corp., Armonk, NY, USA).

## 3. Results

### 3.1. Patient Characteristics

Out of 105 initially enrolled patients, four were excluded because they lost follow-up before completing the 18-month monitoring period. The final analysis included 101 patients; the mean age was 45 years (95% CI: 42–48), and 51.8% were male. Most patients had left-sided colitis (E2, 53.5%), and the median disease duration was 108 months (IQR 60–192). Most patients (87.9%) were receiving mesalazine at the time of colonoscopy. Demographic characteristics and group comparisons are summarized in Table 1.

Baseline characteristics did not significantly differ between the DC and non-DC groups in terms of age, sex, disease extent, duration, or ongoing treatment. In all patients who achieved DC, medical records confirmed the absence of clinical flares and treatment modifications during the three months preceding colonoscopy. In the subgroup of non-DC patients with MES ≥ 1 (n = 41), endoscopic scores were distributed as follows: MES = 1 in 13 patients, MES = 2 in 25, and MES = 3 in 3 individuals. Notably, 13 patients (31%) from this group underwent early modification of medical treatment within one month after colonoscopy due to macroscopic evidence of active inflammation. These therapeutic changes were not considered adverse outcomes in the follow-up analysis. Most of the patients requiring early treatment adjustment had a Total Mayo Score ≥ 6, PRO2 > 2, MES ≥ 2 in all cases, and a GB Score ≥ 4 in 85% (11/13) of cases.

### 3.2. Proportion of Patients Achieving Disease Clearance

**Figure 1:**
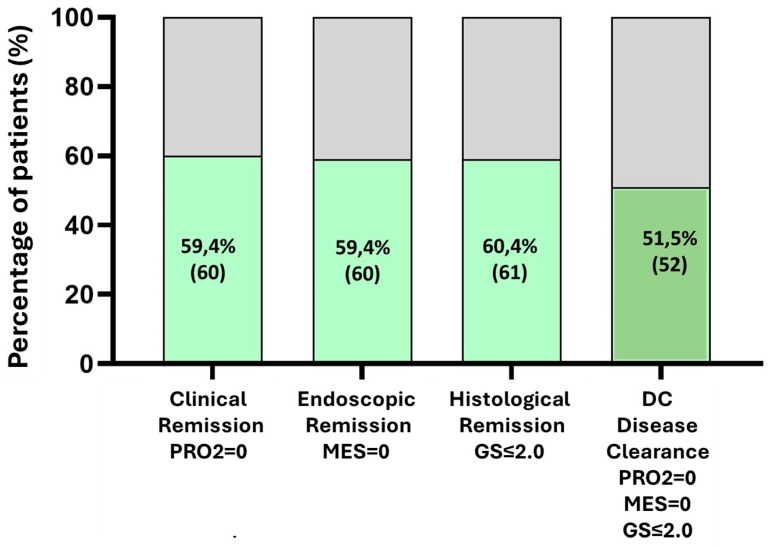
Percentage of patients who achieved disease clearance at colonoscopy.

Of the 101 patients included, 52 (51.5%) met the criteria for disease clearance, defined as simultaneous clinical remission (PRO2 = 0), endoscopic remission (MES = 0), and histological remission (GS ≤ 2). No significant differences in demographics or baseline disease characteristics were observed between the DC and non-DC groups. The proportions of patients achieving each individual component of remission were approximately 60%, with no significant variability (Figure 1).

### 3.3. Clinical Outcomes During Follow-Up

During the 18-month follow-up period, 40 patients (39.6%) experienced a disease flare requiring therapeutic intervention. No hospitalizations or colectomies were reported. The most common treatment modification was corticosteroid initiation (57.6%), including systemic agents such as prednisolone and local action agents as beclometasone. Other interventions included granulocyte apheresis (5%) and thiopurine (5%) or/and anti-TNF therapy (2.5%) initiation.

**Figure 2:**
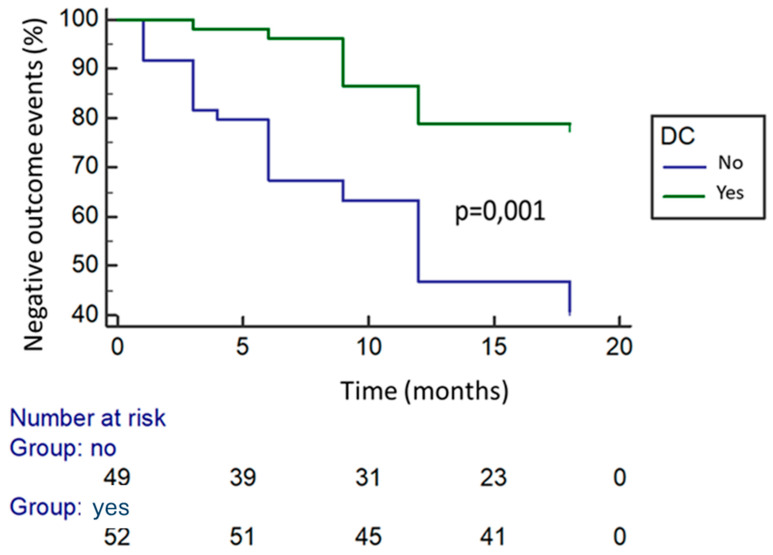
Kaplan–Meier analysis of the risk of developing flares based on disease clearance achievement.

Patients reaching DC had a significantly lower risk of negative outcomes compared to those without DC (non-DC) (21.2% vs. 59.2%; *p* < 0.0001). Kaplan–Meier analysis confirmed this difference (log-rank *p* < 0.001; Figure 2).

### 3.4. Prognostic Value of Each Component of Disease Clearance

**Figure 3:**
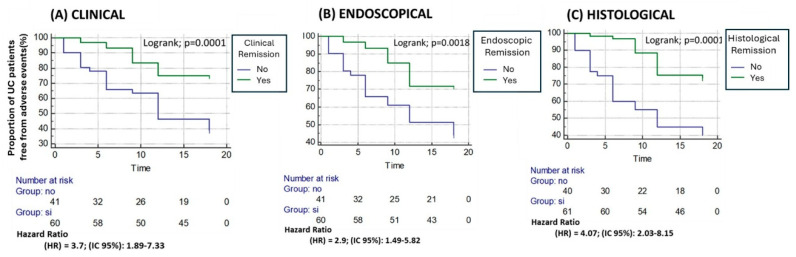
Kaplan–Meier analysis of time to flare based on clinical (**A**), endoscopic (**B**), or histological remission (**C**).

Patients in clinical remission (CR due to PRO2 = 0) at baseline had a lower flare risk than those without CR (25% vs. 61%; *p* < 0.0001), with hazard ratio (HR) 3.7 (95% CI: 1.88–7.33, *p* = 0.0001) (Figure 3A). Similarly, endoscopic remission (ER by MES = 0) was associated with reduced flare risk compared to patients without ER (28.3% vs. 56.1%; *p* = 0.007; HR 2.9, 95% CI: 1.49–5.82; *p* = 0.0018) (Figure 3B). Histological remission (HR due to GS ≤ 2) also provides protection against patients with microscopic inflammation (26.2% vs. 60%; *p* = 0.001; HR 4.07, 95% CI: 2.03–8.15; *p* = 0.001) (Figure 3C).

### 3.5. Complete Remission vs. Isolated Component Remission

**Figure 4:**
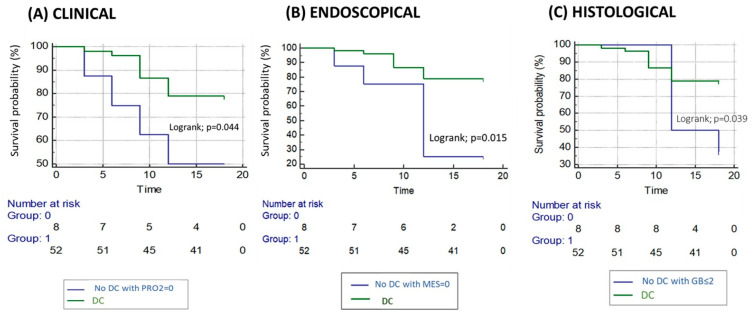
Kaplan–Meier analysis of time to flare in patients achieving clinical (**A**), endoscopic (**B**), or histological (**C**) remission, with or without disease clearance.

Patients who achieved disease clearance (DC) had numerically fewer flares than those with isolated clinical remission (CR) (21.2% vs. 50%; *p* = 0.182), though the difference was not statistically significant. Kaplan–Meier analysis showed a reduced flare risk in the DC group (log-rank *p* = 0.044) (Figure 4A).

Compared to patients with isolated endoscopic remission (ER), those with DC had significantly reduced negative outcomes (21.2% vs. 55.6%; *p* = 0.045), confirmed by survival analysis (log-rank *p* = 0.015) (Figure 4B).

Similarly, patients with DC decreased incidence of flares than those with histological remission (HR) alone (21.2% vs. 55.6%; *p* = 0.045), with Kaplan–Meier analysis supporting a significant difference (log-rank *p* = 0.039) (Figure 4C).

## 4. Discussion

Our study reinforces the growing evidence supporting disease clearance (DC)—defined as simultaneous clinical, endoscopic, and histological remission—as a valuable composite treatment target in ulcerative colitis. In our cohort, patients who achieved DC at baseline had significantly fewer adverse outcomes over 18 months, including reduced rates of relapse and need for therapeutic escalation. These results are consistent with findings from a large multicenter retrospective cohort study by D’Amico et al., which demonstrated that DC, although achieved in only 22% of patients, was independently associated with a markedly reduced risk of hospitalization and surgery over a median 24-month follow-up [15]. Similarly, the UNIFI phase 3 trial showed that patients achieving DC eight weeks after ustekinumab induction were significantly more likely to attain long-term clinical, symptomatic, and quality of life remission during maintenance therapy. At four years, 73.4% of those with early DC achieved symptomatic remission, compared to 53.5% with symptomatic remission alone and 45.1% with neither endpoint [16].

The predictive value of DC has now been confirmed in some real-world studies and post hoc analyses of clinical trials. A recent post hoc analysis of four phase III mesalazine trials reported that approximately 20% of patients achieved DC after 8 weeks of induction, highlighting that this target is achievable even in standard practice [17]. Our findings, with over half of our patients reaching DC, may reflect the impact of early referral to specialized IBD units and the careful selection of candidates undergoing colonoscopy under clinical stability.

Importantly, our study adds granularity by showing that DC confers added prognostic value beyond each of its individual components. While clinical, endoscopic, and histologic remission alone were each associated with improved outcomes, only patients achieving all three simultaneously (i.e., DC) had the most favorable disease course. These observations align with the objectives of the VERDICT trial and subsequent real-world analyses, which aimed to determine the most effective therapeutic target in ulcerative colitis to ensure improved clinical outcomes, including superior flare-free survival, fewer hospitalizations, and lower rates of colectomy [18].

Histological remission, specifically, has gained increasing relevance in ulcerative colitis. Persistent microscopic inflammation despite endoscopic healing has been consistently associated with an increased risk of relapse [19]. However, our findings suggest that histological remission alone may not fully capture the complexity of disease control. In our cohort, patients achieving histological remission without fulfilling DC criteria experienced a higher flare rate compared with those achieving DC. Several explanations may account for this observation. First, histological remission does not necessarily imply complete macroscopic healing, as residual endoscopic activity may persist outside sampled areas due to spatial heterogeneity of inflammation or biopsy sampling limitations. Second, some patients with histological remission but without clinical remission may present persistent symptoms driven by functional disorders, such as IBS-like manifestations, which may lead to treatment escalation or be classified as clinical relapse in real-world practice. By integrating clinical, endoscopic, and histological remission, DC may therefore represent a more robust indicator of complete disease quiescence, minimizing both residual inflammatory activity and functional confounders. This comprehensive control across disease domains likely explains the superior flare-free survival observed in patients achieving DC compared with those achieving histological remission alone. Nonetheless, the importance of including histology in treatment assessment is also reflected in the most recent IOIBD consensus, which incorporates histological endpoints as a highly desirable treatment target in ulcerative colitis [3,20].

Furthermore, the prognostic implications of DC extend beyond surrogate markers. Achieving DC likely reflects more profound immunologic and molecular control of disease activity, potentially preventing irreversible tissue remodeling and fibrotic complications. Indeed, Danese et al. have proposed DC not only as a clinical goal but also as a conceptual bridge toward future targets such as molecular remission [10,13].

Despite its promise, several challenges remain regarding the implementation of DC in routine practice. First, its rigorous criteria may exclude a substantial proportion of patients, particularly those with longstanding or refractory disease. Second, frequent and standardized use of histological scoring systems is not yet universal among clinicians. Third, treatment escalation in patients who are clinically asymptomatic but histologically active continues to raise concerns regarding overtreatment [21]. In these patients, the decision should be individualized, prioritizing treatment escalation in patients with high-risk histologic activity (epithelial neutrophils, ulcerations) and a favorable safety profile. In cases with mild or stable microscopic inflammation, an active surveillance strategy using biomarkers may represent a safer approach [22].

Fecal calprotectin is not currently included in the formal definition of DC, largely due to the lack of a universally accepted cut-off. As highlighted in previous but limited studies [13], reported thresholds for biochemical remission vary widely, although values below 150 µg/g have been pragmatically associated with inactive disease. Future prospective studies should help refine and validate the optimal fecal calprotectin threshold to be incorporated into the DC approach.

In addition, Artificial Intelligence (AI) is increasingly transforming inflammatory bowel disease management, particularly in ulcerative colitis, by enabling more objective and reproducible assessment of disease activity. AI-based tools applied to endoscopic and histological evaluation reduce interobserver variability and improve standardization [23]. These advances may facilitate the real-world implementation of composite targets such as disease clearance by supporting integrated, multimodal assessment of inflammatory activity [24].

Our study has several strengths. First, we applied a strict and standardized definition of DC, incorporating validated measures for clinical (PRO2), endoscopic (MES), and histological (GS) remission. Second, all patients were prospectively followed for 18 months with systematic data collection, ensuring robust outcome assessment. These aspects enhance the internal validity and clinical relevance of our findings.

However, our study also has limitations. Although conducted using a prospective, observational design, it was performed at a single tertiary referral center and included a relatively limited sample size, which may limit the generalizability of the results. In addition, the cohort mainly comprised clinically stable UC patients, with a high proportion treated with mesalazine, potentially underrepresenting those with more severe or refractory disease. Fecal calprotectin was not systematically collected at predefined time points, precluding longitudinal analyses of biomarker kinetics. Finally, the follow-up did not extend beyond 18 months; while this duration was sufficient to capture clinically meaningful outcomes such as disease flares and treatment escalation, it did not permit the evaluation of long-term endpoints, including colectomy or sustained disease modification. Larger, well-designed prospective multicenter studies, incorporating longer follow-up periods and systematic serial biomarker assessment, are warranted to confirm these findings, further define the role of disease clearance, and assess their applicability across broader and more heterogeneous ulcerative colitis populations.

In conclusion, our findings underscore that achieving DC is not only feasible in a significant proportion of patients but also associated with substantial clinical benefit and the potential to modify the natural history of ulcerative colitis. Future prospective studies and trials like VERDICT [17] are expected to further clarify the utility of DC as a therapeutic endpoint and determine whether treat-to-clear strategies should become standard practice in UC management.

## Figures and Tables

**Table 1:** Demographics and clinical features of the ulcerative colitis patients included in the study.

	Global n = 101	DC n = 52	No DC MES = 0 n = 8	No DC MES ≥ 1 n = 41	*p*
Age (years); mean (IC)	45 (42–48)	45 (40–41)	45 (32–59)	44 (39–45)	0.95
Gender (male); n (%)	52 (51.4)	30 (57.7)	3 (37.5)	19 (46.7)	0.23
UC Extension (Montreal)					0.33
E1	15 (14.9)	5 (9.6)	0	10 (24.4)	
E2	54 (53.5)	30 (57.7)	7 (87.5)	17 (31.5)	
E3	32 (31.7)	17 (32.7)	1 (12.5)	14 (43.8)	
Time since UC diagnosis (months); median (IQR)	108 (60–192)	120 (72–192)	72 (48–126)	96 (60–171)	0.44
Type of treatment at the time of colonoscopy; n (%)					
5-ASA	87 (87.9)	43 (82.7)	7 (87.5)	37 (94.9)	0.12
Immunomodulators (thiopurines)	29 (29.3)	13 (25)	2 (25)	14 (35.9)	0.38
Biologicals (anti-TNF)	13 (13.1)	5 (9.6)	0	8 (20.5)	0.37
Treatment change at the time of colonoscopy; n (%)	13 (12.8)	0	0	13 (31)	0.001

DC: Disease Clearance; 5-ASA: mesalazine.

## Data Availability

The data that support the findings of this study are available from the corresponding author upon reasonable request from qualified researchers. All data will be provided following appropriate de-identification procedures and in full compliance with applicable privacy laws, data protection regulations, and requirements for informed consent and anonymization.
